# Enzymes that generate and regulate intracellular persulfides and polysulfides: mechanistic insights and inhibitors

**DOI:** 10.3389/fphys.2026.1764165

**Published:** 2026-02-09

**Authors:** Ko Hirabayashi, Eita Sasaki, Hisashi Ohno, Orie Takayama, Sota Yamada, Kenjiro Hanaoka

**Affiliations:** 1 Graduate School of Pharmaceutical Sciences, Keio University, Tokyo, Japan; 2 Human Biology-Microbiome-Quantum Research Center (WPI-Bio2Q), Keio University, Tokyo, Japan

**Keywords:** mitochondrial respiration, persulfide, polysulfide, protein persulfide, reactive sulfur species, selective inhibitor, sulfide oxidation, transsulfuration pathway

## Abstract

Reactive sulfur species (RSS), which include various persulfides and polysulfides, are generated by multiple enzymes *in vivo* and play critical roles in mammalian physiological processes such as redox signaling, metabolic regulation, radical scavenging and anti-inflammatory responses. Cystathionine *β*-synthase (CBS), cystathionine *γ*-lyase (CSE) and 3-mercaptopyruvate sulfurtransferase (3MST) are well known to mediate endogenous production of hydrogen sulfide (H_2_S), and, together with the mitochondrial isoform of cysteinyl-tRNA synthetase (CARS2), are proposed to be major sources of intracellular persulfides and polysulfides. In mitochondria, enzymes involved in the sulfide oxidation pathway, including sulfide:quinone oxidoreductase (SQOR), persulfide dioxygenase (ETHE1) and thiosulfate sulfurtransferase (TST), also contribute to maintaining and regulating intracellular persulfide levels. Selective inhibitors targeting these enzymes are expected to be powerful tools for elucidating the functions of RSS, as well as having therapeutic potential. In this review, we present a comprehensive overview of these enzymes, focusing on their reaction mechanisms and inhibitors.

## Introduction

**FIGURE 1:**
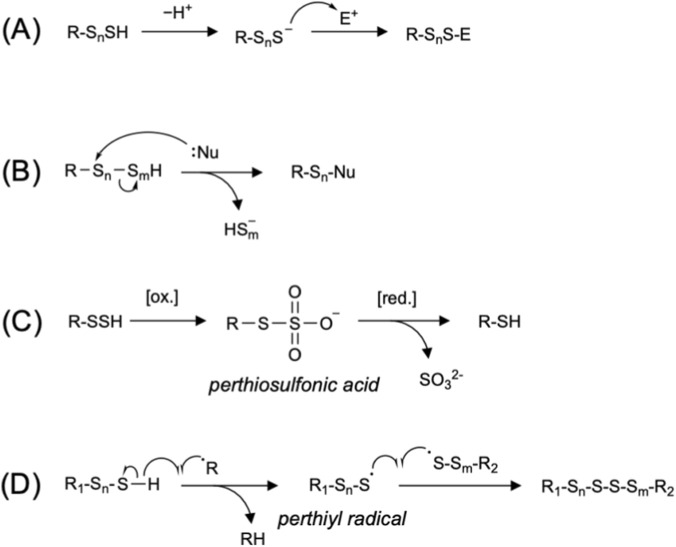
Representative reactions involving hydropersulfides and hydropolysulfides. **(A)** Nucleophilic reactions of hydropersulfides (*n* = 1) or hydropolysulfides (*n* ≥ 2) toward electrophiles (E^+^). **(B)** Electrophilic reactions of hydropersulfides (*n* = 1, m = 1) or hydropolysulfides (n + m ≥ 3) with nucleophiles (:Nu). **(C)** Overoxidation and subsequent reduction of protein persulfides. **(D)** Radical-scavenging reactions of hydropersulfides (*n* = 1, m = 1) or hydropolysulfides (*n* ≥ 2, m ≥ 2). In all panels, *n* and *m*, which represent the number of sulfur atoms, are natural numbers.

Reactive sulfur species (RSS), including (hydro)persulfides and (hydro)polysulfides, are critical mediators in mammalian physiology (Barayeu et al., 2023; Akaike et al., 2024; Pan et al., 2025), playing essential roles in redox signaling (Kasamatsu et al., 2016), metabolic regulation (Akaike et al., 2017; Nishimura et al., 2024), radical scavenging (Wu et al., 2023), anti-inflammatory responses (Tsutsuki et al., 2024), etc. The various biological functions of these molecules arise from their unique chemical properties (Park et al., 2015; Fukuto et al., 2018; Iciek et al., 2022; Switzer, 2023). In general, hydropersulfides/hydropolysulfides serve as stronger nucleophiles than thiols. These species exist largely in a deprotonated anionic form at physiological pH, while most biothiols exist largely in a neutral protonated form (e.g., the p*K*_a_ values of glutathione persulfide (GSSH) and glutathione (GSH) were reported to be 5.45 and 8.94, respectively) (Figure 1A) (Benchoam et al., 2020). In contrast, the hydropersulfides/hydropolysulfides and polysulfides (catenated sulfur), whose terminal sulfurs are both alkylated, are electrophilic and readily react with various nucleophiles such as hydroxide, sulfite, cyanide and thiolate (Figure 1B) (Park et al., 2015). For example, thiolate can nucleophilically attack hydropersulfide to form disulfide and release hydrogen sulfide (H_2_S), which is similar to the reaction of thiolate with sulfenic acid or hydroperoxide (Switzer, 2023). In addition, the persulfidation of protein cysteine functions as an important protective modification to prevent irreversible overoxidation of cysteine residues to sulfinic and sulfonic acids. The S−S bond of the corresponding perthiosulfinic and perthiosulfonic acids can be readily reduced, thereby regenerating the native cysteine (Figure 1C) (Ono et al., 2014). Hydropersulfides/hydropolysulfides also participate in one-electron reactions through perthiyl radicals, which are considerably more stable than the corresponding thiyl radicals (e.g., the S−H bond dissociation energies for persulfide and thiol were reported to be 70 and 92 kcal/mol, respectively) (Bianco et al., 2016; Noguchi et al., 2023). Interestingly, perthiyl radicals rapidly self-recombine to form non-radical products, whereas thiyl radicals tend to abstract a hydrogen atom from other molecules and propagate radical reactions (Figure 1D) (Barayeu et al., 2023; Wu et al., 2023). These radical-scavenging properties of hydropersulfides/hydropolysulfides were recently highlighted in connection with the suppression of lipid peroxidation (Wu et al., 2023). Collectively, the versatile chemical features of RSS enable them to reduce oxidative stress and modulate cellular signaling pathways.

RSS are predominantly produced through reactions catalyzed by a diverse set of sulfur-metabolizing enzymes. Among them, cystathionine *β*-synthase (CBS), cystathionine *γ*-lyase (CSE) and 3-mercaptopyruvate sulfurtransferase (3MST) have long been studied based on their roles in H_2_S biogenesis (Kimura, 2011). Recent studies, however, have broadened our understanding, revealing their potential roles in producing persulfides and polysulfides (Ida et al., 2014). Furthermore, cysteinyl-tRNA synthetase (CARS) has emerged as a novel contributor to intracellular cysteine persulfide (CysSSH) production (Akaike et al., 2017). CARS has also been reported to produce protein persulfides in a translation-coupled manner (Akaike et al., 2017). Mitochondrial enzymes such as sulfide:quinone oxidoreductase (SQOR), persulfide dioxygenase (ETHE1) and thiosulfate sulfurtransferase (TST) further regulate RSS levels in the sulfide oxidation pathway, where H_2_S is converted to thiosulfate and sulfate (Libiad et al., 2014; Hanna et al., 2023).

Despite the growing understanding of the physiological roles of RSS, the precise contribution of each enzyme to specific biological events remains elusive. Although genetic methods (i.e., gene knockout and knockdown) are powerful approaches, they often activate compensatory pathways, making it difficult to determine the specific role of each enzyme (Marutani and Ichinose, 2020; Zainol et al., 2023). In contrast, selective inhibitors targeting each enzyme can block its activity at defined time points and in a dose-dependent manner, enabling more refined experimental control. Small molecule inhibitors may also have therapeutic potential (Whiteman et al., 2011). This review aims to provide a comprehensive overview of the enzymatic reaction mechanisms underlying the biogenesis and regulation of persulfides and polysulfides, and also to highlight key inhibitors together with their modes of action.

## Enzymes and reaction pathways

**FIGURE 2:**
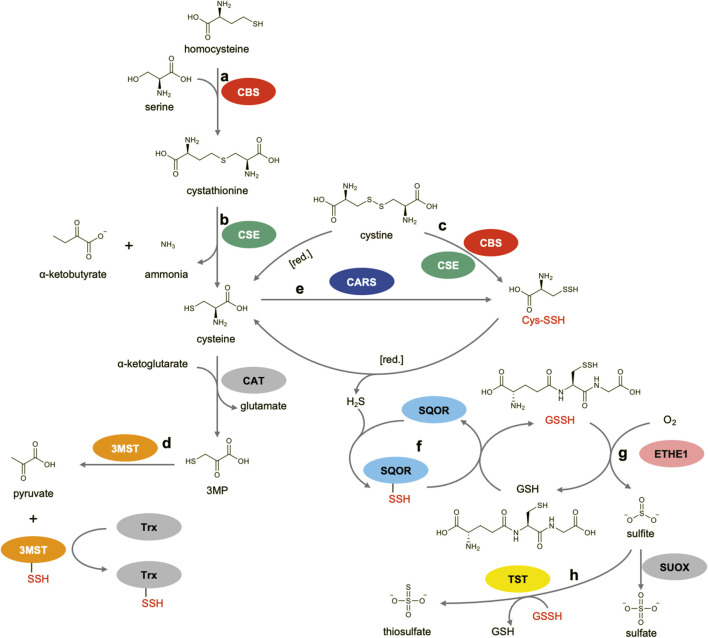
Enzymatic pathways involved in the production and regulation of RSS. **(a)** CBS-catalyzed reaction in the transsulfuration pathway. **(b)** CSE-catalyzed reaction in the transsulfuration pathway. **(c)** Possible CBS/CSE-catalyzed reaction for generation of CysSSH from cystine. **(d)** 3MST-catalyzed reaction. **(e)** CARS-catalyzed reaction for the generation of CysSSH from two molecules of cysteine. **(f)** SQOR-catalyzed reaction in the mitochondrial sulfide oxidation pathway. **(g)** ETHE1-catalyzed reaction in the mitochondrial sulfide oxidation pathway. **(h)** TST-catalyzed reaction in the mitochondrial sulfide oxidation pathway.

Biogenesis and regulation of persulfides and polysulfides are orchestrated by several key enzymes (Figure 2). CBS and CSE, both of which are pyridoxal 5′-phosphate (PLP)-dependent enzymes, function sequentially in the transsulfuration pathway, converting homocysteine and serine into cystathionine and subsequently cysteine (Figures 2 a,b) (Sbodio et al., 2019). In addition to these canonical reactions, both enzymes have been proposed to produce H_2_S by utilizing alternative substrates, namely, a combination of cystine and homocysteine for CBS and either cysteine or homocysteine for CSE (Sbodio et al., 2019). In 2014, Ida et al. reported that CBS and CSE can also use cystine as a preferential substrate and produce cysteine persulfide (CysSSH) *in vitro* (Figure 2c) (Ida et al., 2014). Furthermore, mass spectrometry (MS)-based metabolomic analysis revealed that the concentrations of low-molecular-weight persulfides/polysulfides, including CysSSH and GSSH, in A549 cells were significantly increased or decreased when the corresponding enzymes were overexpressed or knocked down, respectively (Ida et al., 2014). However, the same group later revisited these findings and proposed a revised view of the roles of CBS and CSE *in vivo*. Rather than directly producing CysSSH formation from cystine, these enzymes are now thought to contribute primarily to cysteine production via the transsulfuration pathway, and it was suggested that CARS, which can use cysteine as a substrate to produce CysSSH, is a more plausible major contributor to intracellular CysSSH biogenesis (Akaike et al., 2017). One line of evidence supporting this view is that intracellular cystine concentrations (sub-micromolar to low micromolar) are far below the *K*_m_ value of CSE for cystine (>200 µM), making efficient CysSSH formation from cystine unlikely under physiological conditions. Nevertheless, CBS and CSE may still produce CysSSH under pathophysiological conditions in which the intracellular cystine concentration is markedly elevated.

3MST is another enzyme known to be involved in H_2_S biogenesis, and is also reported to generate persulfidated species (Kimura et al., 2017; Pedre and Dick, 2021). It is predominantly localized in mitochondria and functions as a sulfurtransferase that converts 3-mercaptopyruvate, which is produced from cysteine by cysteine aminotransferase (CAT), into pyruvate (Figure 2d) (Nag et al., 2006; Kimura, 2015). 3MST transfers the sulfur of 3-mercaptopyruvate to its active-site cysteine, affording a 3MST-bound persulfide intermediate. This intermediate subsequently transfers its sulfur to a thiophilic acceptor molecule, such as thioredoxin (Trx) (Mikami et al., 2011). In 2017, Kimura et al., reported that 3MST in a mouse brain cell suspension could produce various RSS including hydrogen disulfide (H_2_S_2_), hydrogen trisulfide (H_2_S_3_), CysSSH, and GSSH in addition to H_2_S (Kimura et al., 2017). Therefore, this enzyme may contribute to the *in vivo* generation of protein persulfides, as well as low-molecular-weight persulfides/polysulfides.

In 2017, Akaike et al. reported a study that drew increased attention to RSS. They showed that CARS, an enzyme canonically known for catalyzing the attachment of cysteine to its cognate transfer RNA (tRNA), is the major source of endogenous persulfides/polysulfides (Akaike et al., 2017). They discovered that CARS catalyzes a novel PLP-dependent reaction to produce CysSSH using two molecules of cysteine as substrates (Figure 2e). They also found that the enzyme may utilize CysSSH as a substrate for aminoacylation, thereby enabling its incorporation into nascent polypeptides during translation (Akaike et al., 2017). Interestingly, knockout (KO) of the mitochondrial isoform of CARS (CARS2) in HEK293T cells resulted in an approximately two-thirds reduction in intracellular CysSSH levels compared with wild-type (WT) cells. In addition, CBS and CSE knockdown in CARS2 KO cells suppressed the intracellular cysteine levels, but did not lead to a further reduction in the CysSSH levels, suggesting that CARS2 is the major enzyme responsible for CysSSH production (Akaike et al., 2017). In 2023, Zainol Abidin et al. further supported this conclusion by performing an extensive sulfur metabolome analysis in CBS/CSE/3MST triple-KO mice (Zainol et al., 2023). Their results showed that CysSSH levels in the triple-KO mice were not significantly changed compared with those of WT mice. In contrast, the CysSSH levels in liver and lung tissues of CARS2-deficient heterozygous mice were reduced by more than 50% compared with those of WT mice, highlighting the principal role of CARS2 in CysSSH biogenesis (Zainol et al., 2023).

In the mitochondrial sulfide oxidation pathway, SQOR, a flavin adenine dinucleotide (FAD)-dependent enzyme, initiates H_2_S oxidation coupled with the reduction of coenzyme Q (CoQ or ubiquinone) (Landry et al., 2021). During catalysis, FAD is reduced to its dihydrogenated form (FADH_2_), which subsequently reduces CoQ. Electrons from the reduced CoQ then enter the mitochondrial electron transport chain at complex III. In the SQOR-catalyzed reaction, sulfur of H_2_S is accepted by GSH to generate GSSH under physiological conditions (Figure 2f) (Landry et al., 2021). The released GSSH is then transferred to the downstream enzyme ETHE1, which contains an iron atom in its active site. It catalyzes the conversion of GSSH to GSH and sulfite (Figure 2g) (Kabil and Banerjee, 2012). Subsequently, TST, which belongs to the sulfurtransferase family, like 3-MST, converts sulfite to thiosulfate using another molecule of GSSH as a sulfur donor (Figure 2h) (Buonvino et al., 2022). Alternatively, sulfite is further oxidized to sulfate by sulfite oxidase (SUOX) (Fu et al., 2025). Although H_2_S is a toxic gas that binds to the heme of cytochrome c oxidase and inhibits mitochondrial respiration, it also serves as an electron donor for the mitochondrial electron transport chain (Bouillaud and Blachier, 2011). Therefore, SQOR and its downstream enzymes play important roles in energy generation by regulating H_2_S and RSS (primarily GSSH) levels.

## Reaction mechanisms and inhibitors

### CBS

**FIGURE 3:**
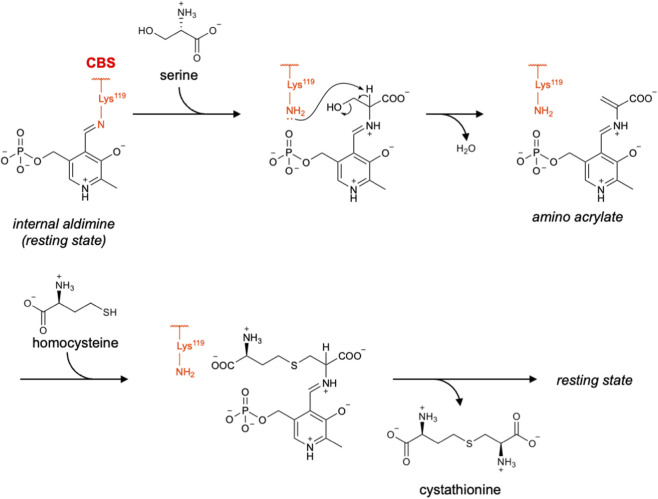
Proposed catalytic mechanism of CBS.

CBS is a PLP-dependent *β*-replacement enzyme in the transsulfuration pathway. It utilizes homocysteine and serine to generate cystathionine (Banerjee et al., 2003). In the canonical CBS-catalyzed reaction, serine first forms an external aldimine with PLP. Subsequently, a *β*-elimination reaction occurs, releasing a water (H_2_O) molecule and yielding an aminoacrylate intermediate. This is the key intermediate in the CBS reaction and was directly observed by time-resolved spectroscopy (Yadav et al., 2012). Next, the thiolate of homocysteine nucleophilically attacks this aminoacrylate, leading to cystathionine formation (Figure 3) (Yadav et al., 2012; Banerjee and Zou, 2005).

Mammalian CBS is an unusual PLP enzyme that contains a heme cofactor and *S*-adenosyl-L-methionine (SAM). Although the C-terminal SAM-binding site is located away from the catalytic center, it was revealed that SAM binding induces a structural rearrangement of the catalytic core that relieves autoinhibition and stabilizes an activated conformation (McCorvie et al., 2014; Ereño-Orbea et al., 2014). Therefore, SAM allosterically enhances the enzyme activity (Janosík et al., 2001; Prudova et al., 2006). The crystal structure of human CBS had been determined only after deletion of the regulatory loop (aa 515–525) (McCorvie et al., 2014; Ereño-Orbea et al., 2014), but McCorvie et al. recently visualized the structure of full-length human CBS using cryo-electron microscopy (cryo-EM) (McCorvie et al., 2024). Interestingly, the full-length enzyme assembles into higher-order oligomers forming helical filaments. The conformation of these filaments was shown to change upon SAM binding, from three CBS dimers per turn to two. This conformational change results in the removal of the steric block imposed by the regulatory domains on the active site of the catalytic core (McCorvie et al., 2024).

**FIGURE 4:**
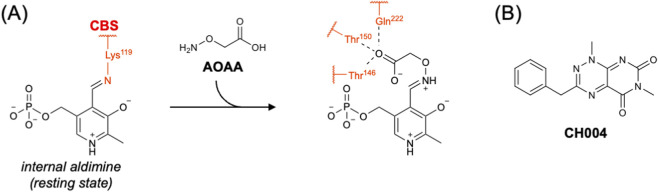
Inhibitor of CBS. **(A)** Chemical structure of AOAA and its interactions with active-site residues of CBS. **(B)** Chemical structure of CH004.

Historically, aminooxyacetic acid (AOAA) has been the most commonly used inhibitor of CBS (Whiteman et al., 2011; Asimakopoulou et al., 2013; Zuhra et al., 2020). Although AOAA has been suggested to act as an irreversible inhibitor that forms a dead-end oxime complex with PLP (Zuhra et al., 2020), Petrosino et al. recently demonstrated that the catalytic activity of AOAA-modified CBS can be rescued in the presence of high concentrations of serine (Petrosino et al., 2022). They also reported the crystal structure of human CBS complexed with AOAA, in which the oxime intermediate is stabilized by a newly formed hydrogen-bond network involving Thr146, Thr150, and Gln222 (Figure 4A) (Petrosino et al., 2022). However, it should be noted that AOAA is a broad-spectrum inhibitor of PLP-dependent enzymes. For example, it shows similar inhibitory activity toward CSE and CBS with half-maximal inhibitory concentration (IC_50_) values of 1.1 µM and 8.5 µM, respectively (Asimakopoulou et al., 2013). Therefore, its limited selectivity must be considered when interpreting cellular or *in vivo* experiments.

To overcome this limitation, numerous high-throughput screening efforts to identify CBS inhibitors have been conducted, but a truly CBS-selective compound has yet to be discovered (Ascenção and Szabo, 2022; Sasaki et al., 2023). One inhibitor, CH004, identified by Wang et al. in 2018, exhibits an IC_50_ value of 1 μM for CBS and shows approximately 30-fold higher inhibitory activity toward CBS than toward CSE (Figure 4B) (Wang et al., 2018). They also demonstrated that CH004 binds reversibly to CBS and determined the dissociation constant (*K*_d_) to be 0.6 μM. Although the structure of the CBS–CH004 complex has not yet been reported, Q222A mutation largely abolishes the inhibitory activity of CH004, suggesting that Gln222 is a key residue for its binding. A potential issue with CH004 is that it also possesses direct H_2_S-scavenging activity (Wang et al., 2018). In addition, several other targets unrelated to CBS have been suggested by later studies (Ascenção and Szabo, 2022).

### CSE

**FIGURE 5:**
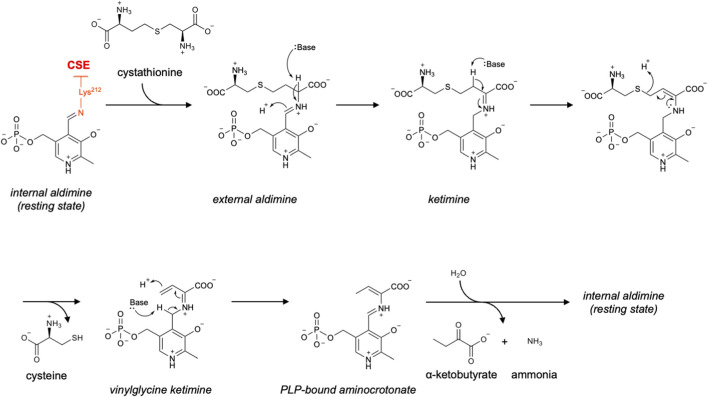
Proposed catalytic mechanism of CSE.

CSE is another PLP-dependent enzyme in the transsulfuration pathway. It catalyzes the breakdown of cystathionine into cysteine, *α*-ketobutyrate, and ammonia (Sbodio et al., 2019; Stipanuk and Ueki, 2011). In the resting state, PLP is covalently bound to an active-site lysine residue (Lys212) as an internal aldimine. Cystathionine first enters the active site to form an external aldimine with PLP. It is then tautomerized to ketimine and subsequently undergoes cleavage of the Cγ–S bond to release cysteine. The remaining vinylglycine ketimine intermediate is tautomerized to the PLP-bound aminocrotonate, which is subsequently hydrolyzed to release *α*-ketobutyrate and ammonia. Finally, Lys212 forms a Schiff base with PLP to restore the resting state (Figure 5) (Chiku et al., 2009; Sun et al., 2009; Huang et al., 2010).

In addition to the canonical *γ*-lyase reaction, CSE also catalyzes *β*-elimination reactions with various substrates including cysteine and cystine (Chiku et al., 2009). Namely, it has potential for *in vivo* generation of H_2_S and CysSSH from cysteine and cystine, respectively. The former reaction has been reported to be important particularly in the cardiovascular system (Zhao et al., 2014; Kimura, 2014). The latter reaction has been analyzed in detail using rat liver CSE (Yamanishi and Tuboi, 1981), and Ida et al. reported that human CSE can also catalyze this reaction at least *in vitro* (Ida et al., 2014). In contrast, more recent studies using CBS/CSE/3MST triple-KO mice suggest that mitochondrial CARS2 is the major contributor to both H_2_S and CysSSH production *in vivo* (Zainol et al., 2023). In 2023, Araki et al. reported that persulfidation of Cys136 in rat CSE suppresses its *β*-lyase activity to generate CysSSH from cystine (Araki et al., 2023). Similarly, in 2024, Jia et al. showed that persulfidation of Cys137 in human CSE (equivalent to Cys136 in rat CSE) decreases the *β*-lyase activity to generate H_2_S from cysteine (Jia et al., 2024). Both studies suggest that persulfidation of this redox-sensitive cysteine residue functions as a negative feedback mechanism regulating the production of H_2_S or CysSSH. The relative contributions of CSE and CARS to H_2_S and CysSSH biogenesis *in vivo* remain to be fully clarified.

**FIGURE 6:**
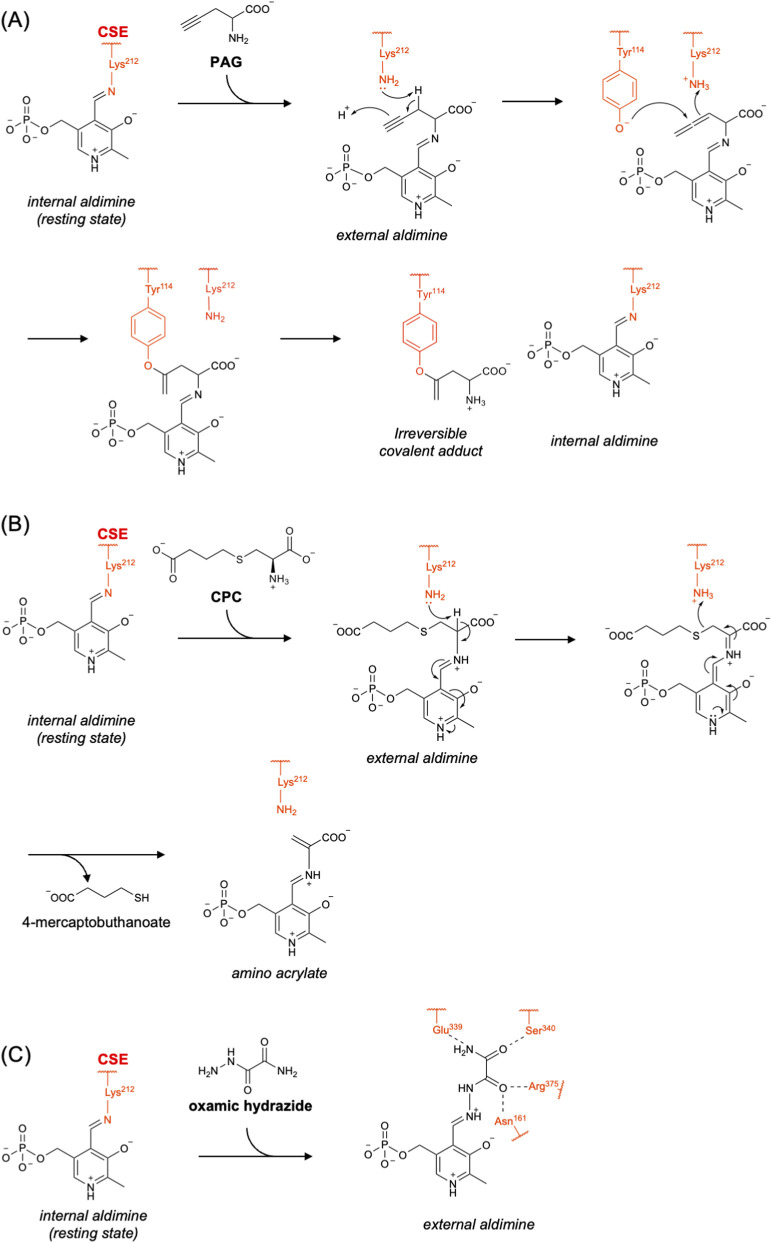
Inhibitors of CSE. Chemical structures and inhibitory mechanisms of **(A)** PAG, **(B)** CPC and **(C)** oxamic hydrazide.

Propargylglycine (PAG) is an irreversible inhibitor of CSE (Whiteman et al., 2011; Asimakopoulou et al., 2013; Abeles and Walsh, 1973). It first forms an external aldimine with PLP, like other CSE substrates. Lys212 has been proposed to deprotonate the *β*-position of PAG, yielding a reactive allene intermediate. This intermediate is nucleophilically attacked by the active site tyrosine residue (Tyr114) to form a covalent vinylether adduct, leading to inhibition of the enzyme (Figure 6A) (Sun et al., 2009; Yadav et al., 2019). Sun et al. reported the crystal structure of the CSE complex with PAG, confirming a covalent bond between Tyr114 and Cγ of PAG (Sun et al., 2009). Although high selectivity of PAG for CSE over CBS has been reported (i.e., an IC_50_ of 40 μM for the CSE-catalyzed reaction using 1 mM of cysteine as the substrate, with no detectable inhibition of CBS even at 10 mM PAG), it also inhibits several other PLP enzymes, such as aspartate aminotransferase, alanine aminotransferase, and methionine *γ*-lyase (Asimakopoulou et al., 2013; Sun et al., 2009). In 2019, Yadav et al. showed that the extent of PAG’s inhibitory activity toward CSE, with cysteine as the substrate, depends on the preincubation time of the inhibitor with the enzyme as well as on the concentration of cysteine (Yadav et al., 2019). They proposed that cysteine competitively interferes with PAG binding to the enzyme, thereby preventing PAG from completing the formation of the dead-end complex with Tyr114. Therefore, the effective concentration of PAG required for CSE inhibition may vary substantially across tissues with different intracellular cysteine levels (Yadav et al., 2019).

As an alternative CSE inhibitor, Yadav et al. identified a cystathionine analog, *S*-3-carboxypropyl-L-cysteine (CPC), with a reversible inhibitory mechanism (Yadav et al., 2019). It suppresses both cystathionine cleavage and H_2_S production by human CSE with inhibition constant (*K*ᵢ) values of 50 µM and 180 μM, respectively. They also demonstrated that it does not exhibit inhibitory activity toward other PLP-dependent enzymes, CBS and CAT, or the H_2_S-producing enzyme 3MST at concentrations up to 5–10 mM. In addition, the crystal structure of the human CSE complex with CPC has been reported, confirming the presence of the PLP-bound amino acrylate intermediate (Figure 6B) (Yadav et al., 2019).

Our group recently identified oxamic hydrazide as a potent CSE inhibitor by screening a large chemical library of 161,600 compounds (Echizen et al., 2023). It forms a Schiff base with the active-site PLP of CSE and exhibits an IC_50_ value of 13 µM with high selectivity over other PLP-dependent enzymes including CBS, alanine aminotransferase and methionine *γ*-lyase. The high selectivity of oxamic hydrazide toward CSE was rationalized based on the crystal structure of the rat CSE complex with this inhibitor. In this structure, the PLP-bound oxamic hydrazide is stabilized through hydrogen bonds formed with the active-site residues Glu339, Ser340, Arg375, and Asn161 (Figure 6C). Importantly, these amino acids are conserved in human CSE, and inhibitory activity of oxamic hydrazide toward human CSE has been confirmed in cellular assays using HEK293 cells overexpressing this enzyme (Echizen et al., 2023).

### 3MST

**FIGURE 7:**
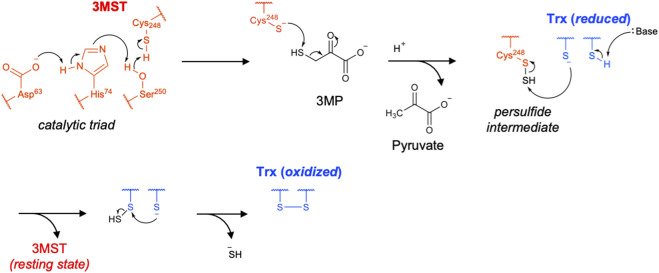
Proposed catalytic mechanism of 3MST.

Catalysis by 3MST involves a two-step ping–pong mechanism. The active-site cysteine first attacks 3-mercaptopyruvate to form an enzyme-bound cysteine persulfide intermediate while releasing pyruvate. Subsequently, the outer sulfur atom of this persulfide is transferred to thiophilic acceptors (Pedre and Dick, 2021). The crystal structure of human 3MST reveals that 3-mercaptopyruvate is positioned adjacent to Arg188 and Arg197, to which it is linked through hydrogen bonds. A Ser–His–Asp triad has been proposed to facilitate thiolate formation at Cys248, enabling nucleophilic attack on the sulfur atom of 3-mercaptopyruvate (Figure 7) (Yadav et al., 2013).

So far, various thiol-containing biomolecules such as Trx, dihydrolipoic acid, cysteine and molybdenum cofactor synthesis protein 3 (MOCS3) have been proposed and investigated as sulfur acceptors (Kimura et al., 2017; Mikami et al., 2011; Yadav et al., 2013; Fräsdorf et al., 2014; Yadav et al., 2020). Yadav et al. demonstrated that Trx is the physiologically preferred sulfur acceptor based on detailed kinetic analyses (Yadav et al., 2013; Yadav et al., 2020). They determined that the Michaelis constant (*K*_m_) for Trx (2.5 μM) is reasonably low compared with physiological Trx concentrations (1–20 μM). Further, the catalytic efficiency (*k*_cat_/*K*_m_) of human 3MST toward Trx is at least 1,000-fold higher than that toward dihydrolipoic acid or cysteine. Upon accepting sulfur from 3MST, Trx undergoes intramolecular attack by its vicinal thiol, forming an internal disulfide bond and thereby yielding oxidized Trx and releasing H_2_S (Pedre and Dick, 2021; Yadav et al., 2013). In contrast, reduced Trx interacts with 3MST and causes substrate inhibition, resulting in an increased *K*_m_ for 3-mercaptopyruvate (Yadav et al., 2020). Therefore, under oxidative conditions, where the level of reduced Trx is low, the sulfur-transfer activity of 3MST may be enhanced, leading to the increased production of low-molecular-weight RSS, such as CysSSH (Yadav et al., 2020).

**FIGURE 8:**
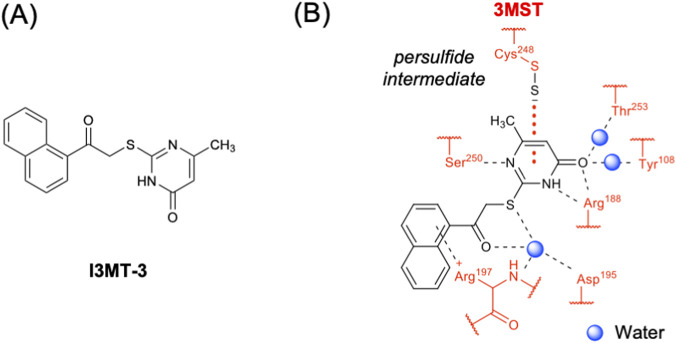
Inhibitor of 3MST. **(A)** Chemical structure of I3MT-3. **(B)** Interactions of I3MT-3 with the active-site residues of 3MST.

In 2017, our group identified a potent and selective inhibitor of 3MST (Hanaoka et al., 2017). This compound, later referred to as I3MT-3 or HMPSNE (2-[(4-hydroxy-6-methylpyrimidin-2-yl)sulfanyl]-1-(naphthalen-1-yl)ethan-1-one), exhibits an IC_50_ value of 2.7 μM toward mouse 3MST and shows high selectivity over rat CBS, rat CSE, and bovine rhodanese (Hanaoka et al., 2017). Augsburger et al. determined that the IC_50_ value of I3MT-3 toward human 3MST is 13.6 μM (Augsburger et al., 2020). The crystal structure of the rat 3MST complex with I3MT-3 revealed a unique binding mode, in which the persulfidated anion at the active site cysteine (Cys248) has a long-distance electrostatic interaction (>3.45 Å) with the positively charged carbonyl carbon of the pyrimidone ring. Direct hydrogen bonds with Arg188 and Ser250, as well as water-mediated hydrogen bonds with Tyr108, Glu195, Arg197 and Thr253 were also observed (Figure 8) (Hanaoka et al., 2017). In addition, isothermal titration calorimetry demonstrated that I3MT-3 binds tightly and selectively to the persulfidated form of 3MST with a *K*_d_ value of 0.5 μM, whereas no binding was detected with the reduced form (Hanaoka et al., 2017). I3MT-3 has become a standard pharmacological probe for suppressing 3MST-dependent bioenergetics and tumor cell proliferation (Rao et al., 2023). For example, it has been used in a murine colon cancer cell line (CT26) (Augsburger et al., 2020), a human endothelial cell line (EA.hy926) (Abdollahi Govar et al., 2020), a human colon cancer cell line (HCT116) (Ascenção et al., 2022a), human colonic epithelial cell organoids (Ascenção et al., 2022b) and porcine coronary artery segments (Almaheize et al., 2025). In addition to the inhibitory activity toward 3MST, a recent study by Otani et al. demonstrated that I3MT-3 directly inhibits caspase-1, suppressing IL-1β release and pyroptosis induced by multiple effectors (Otani et al., 2025).

### CARS

CARS catalyzes the formation of cysteinyl aminoacyl-tRNA^Cys^ (Cys-tRNA^Cys^) through ATP-dependent activation of L-cysteine, yielding cysteinyl adenylate (Cys-AMP), followed by transfer of cysteine to the 3′-terminus of tRNA^Cys^ (Newberry et al., 2002; Hauenstein et al., 2004). In addition to this canonical role, Akaike et al. reported in 2017 that CARS also has cysteine persulfide synthase (CPERS) activity, producing CysSSH in a PLP-dependent manner (Akaike et al., 2017). They determined the *K*_m_ of *E. coli* CARS for this reaction to be 7.4 μM, which is sufficiently low relative to intracellular cysteine concentrations (100–1000 μM) (Akaike et al., 2017; Fujii et al., 2019). LC-MS/MS analysis of the CARS-catalyzed CysSSH formation using stable-isotope-labeled cysteine further indicated that sulfur is transferred from one cysteine molecule to another. Moreover, extensive mutational analyses using *E. coli* CARS showed that the lysine residues within the ^73^KIIK^76^ and ^266^KMSK^269^ motifs are important for CPERS activity (but not for aminoacylation activity) and likely serve as PLP binding sites (Akaike et al., 2017). Interestingly, these KIIK and KMSK motifs are highly conserved across diverse species, including mammals, supporting their functional relevance in CPERS activity (Akaike et al., 2017).

In mammals, there are two isoforms of CARS: CARS1, which is localized in the cytosol, and CARS2, which is localized in the mitochondria (Hallmann et al., 2014). Both isoforms possess CPERS activity (Akaike et al., 2017; Fujii et al., 2019). The study by Akaike et al., together with subsequent *in vivo* and metabolomic analyses, has strengthened the view that CARS2 is the principal source of intracellular persulfides (Akaike et al., 2017; Nishimura et al., 2024; Zainol et al., 2023). CARS2-derived CysSSH has also been proposed to receive electrons from components of the mitochondrial electron transport chain and thereby produce H_2_S (Akaike et al., 2017). This model implies that CARS2 may contribute not only to persulfide biogenesis but also to mitochondrial respiration, functioning upstream of the sulfide oxidation pathway involving SQOR, ETHE1 and TST. However, the precise catalytic mechanism underlying CARS-mediated persulfide formation remains unresolved (Kasamatsu and Ihara, 2021; Ogata et al., 2023; Vignane and Filipovic, 2023). To date, no inhibitor specifically targeting the CPERS activity of CARS has been reported.

### SQOR

SQOR is an inner mitochondrial membrane-associated flavoprotein that catalyzes the two-electron oxidation of H_2_S using CoQ as an electron acceptor and a low-molecular-weight sulfur acceptor, most likely GSH (Landry et al., 2021). The first crystal structure of human SQOR was reported in 2019 by Jackson et al. (2019), who found that two redox-active cysteine residues (Cys201 and Cys379) are bridged by a sulfane sulfur, forming an internal cysteine trisulfide species. This intermediate was previously proposed to represent an inactive form of the enzyme, which may be slowly formed in the absence of an external sulfur acceptor (Jackson et al., 2012). In contrast, Landry et al. proposed in the same year that the trisulfide species is the catalytically active form and represents the resting state of the enzyme, based on detailed structural and kinetic experiments (Landry et al., 2019). In agreement with this proposal, disruption of the trisulfide bridge by cyanide treatment led to destabilization and inactivation of the enzyme (Landry et al., 2020). Landry et al. also predicted, based on computational modeling and molecular dynamics simulations, that nucleophilic addition of sulfide to the trisulfide is approximately 10^5^-fold faster than that to the disulfide within the enzyme (Landry et al., 2020). In fact, the *k*_cat_/*K*_m_ of SQOR toward H_2_S was estimated to be of the order of 10^7^ M^-1^s^-1^ (Jackson et al., 2012), which is more than10^7^-fold higher than the rate constant for sulfide addition to disulfide in solution at pH 7.4 and 25 °C (0.6 M^-1^s^-1^) (Cuevasanta et al., 2015).

**FIGURE 9:**
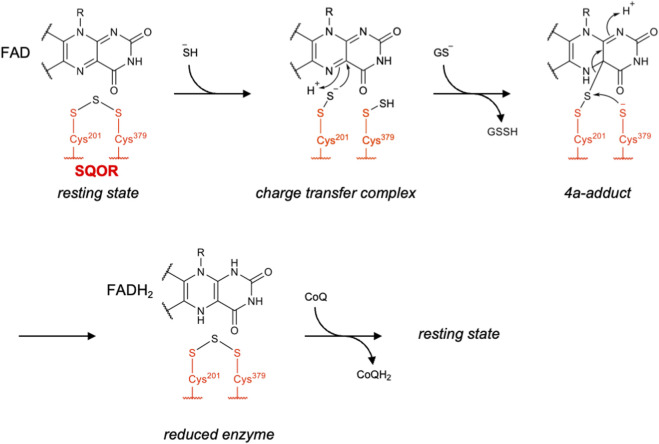
Proposed catalytic mechanism of SQOR.

In the proposed catalytic cycle, sulfide first attacks the trisulfide bridge of the enzyme, generating persulfides at both Cys201 and Cys379. Next, the persulfide at Cys201 attacks the C4a position of FAD, whereas the sulfur on Cys379 is transferred to an external sulfur acceptor. FAD is then fully reduced to FADH_2_ upon nucleophilic attack by Cys379 on the disulfide linkage between Cys201 and the FAD adduct, thereby reforming the initial trisulfide bridge. Finally, the electrons from FADH_2_ are transferred to CoQ, resulting in regeneration of oxidized FAD (Figure 9) (Landry et al., 2021; Landry et al., 2019). Early kinetic analyses suggested that sulfite is a preferred sulfur acceptor in this reaction, as it exhibits a high *k*_cat_/*K*_m_ (2.9 × 10^7^ M^-1^ s^-1^) (Jackson et al., 2012). However, due to the low intracellular concentration of free sulfite (<20 nM) compared with its *K*_m_ value (∼200 μM), it is now considered that the physiologically most relevant sulfur acceptor is GSH. GSH exists at a concentration of 1–10 mM in cells and shows a *K*_m_ of 8 mM and a *k*_cat_/*K*_m_ of 1.6 × 10^4^ M^-1^ s^-1^ in the SQOR-catalyzed reaction (Landry et al., 2021; Landry et al., 2017).

**FIGURE 10:**
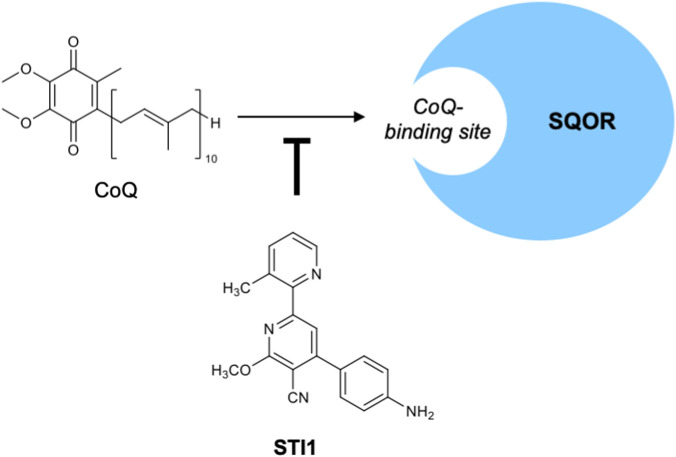
Inhibitor of SQOR. Chemical structure of STI1 and its inhibitory mechanism, i.e., competition with CoQ binding to SQOR.

For many years, no selective small-molecule inhibitor of SQOR was known, which limited pharmacological studies of this enzyme. In 2021, however, Baugh et al. identified the first potent inhibitors of human SQOR (Baugh et al., 2021). They screened 41,000 compounds and synthesized 120 analogs to study the structure-activity relationships. As a result, a 2,4-diphenylpyridine derivative, later named STI1 (Jackson et al., 2022), was found to be the best inhibitor of human SQOR, exhibiting an IC_50_ value of 29 nM with suitable physicochemical properties and good cell permeability (Figure 10) (Baugh et al., 2021). Docking simulations of STI1 to a ligand-free SQOR crystal structure indicated that it binds within the CoQ-binding pocket (Baugh et al., 2021). Steady-state kinetic studies further confirmed that STI1 competitively inhibits CoQ binding, thereby blocking the catalytic cycle of SQOR (Jackson et al., 2022). STI1 exhibited high selectivity for the SQOR-catalyzed reaction compared with other CoQ-dependent reactions; specifically, the off-target IC_50_ values for the respiratory complexes I, II, and III, dihydroorotate dehydrogenase, and electron transferring flavoprotein:ubiquinone oxidoreductase were all at least 1,000-fold higher than the IC_50_ for SQOR (Jackson et al., 2022). Moreover, low cytotoxicity of STI1 was demonstrated in a rat ventricular cardiomyoblast cell line (H9c2 cells; half-maximal cytotoxic concentration, CC_50_, of 56 μM) and in neonatal rat ventricular cardiomyocytes (CC_50_ of 26 μM). Finally, STI1 was applied in a pressure-overload-induced heart failure model in mice. Pharmacological inhibition of SQOR with STI1 preserved cardiac function and prevented adverse remodeling. These results indicate that SQOR is an attractive target for therapeutic modulation of endogenous H_2_S signaling (Jackson et al., 2022).

### ETHE1

ETHE1 is a mononuclear non-heme iron persulfide dioxygenase localized in the mitochondrial matrix. It catalyzes the molecular oxygen (O_2_)-dependent oxidation of GSSH, yielding sulfite and GSH. Thus, ETHE1 catalyzes the second step of the mitochondrial H_2_S oxidation pathway downstream of SQOR. The crystal structure of human ETHE1 was reported in 2015 by Pettinati et al. (Pettinati et al., 2015). The enzyme harbors a mononuclear Fe(II) center coordinated by His79, His135 and Asp154, together with three water molecules, resulting in an octahedral geometry. It has a substrate-binding channel leading to the active site that is sufficiently large to accommodate GSSH.

**FIGURE 11:**
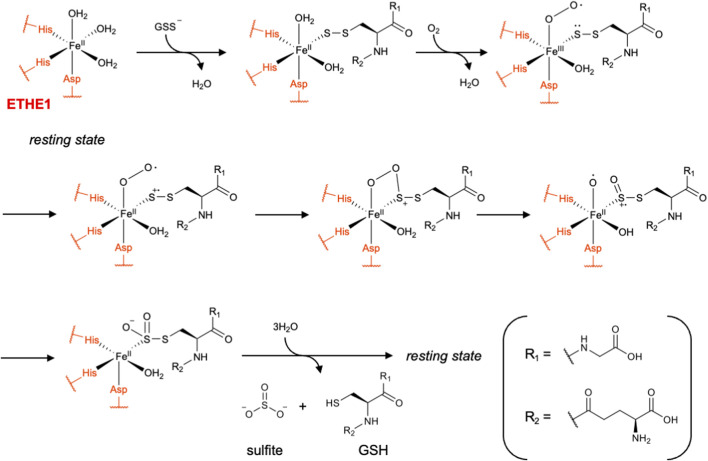
Proposed catalytic mechanism of ETHE1.

In 2016, Lin et al. proposed a mechanism for GSSH oxidation catalyzed by ETHE1 based on quantum mechanics/molecular mechanics (QM/MM) calculations (Lin et al., 2016). GSSH first displaces one water ligand and positions its terminal sulfur for coordination to Fe(II). Subsequent O_2_ binding yields an Fe(III)-superoxo species that is in resonance with a formulation in which the coordinated sulfur bears a partial radical character. Recombination of this superoxo/sulfur radical pair produces a cyclic peroxo–sulfur intermediate, which then undergoes O–O bond homolysis to give a sulfoxy cation and an Fe(II)-bound oxo species. Hydrolysis of the perthiosulfinic intermediate (GS–SO_2_H) by an iron-bound water molecule releases sulfite and GSH, returning the enzyme to its resting state (Figure 11) (Lin et al., 2016; Kabil et al., 2018). Mutation of Cys247, located near the active-site iron, abolished the dioxygenase activity, indicating that this residue plays an important role in the catalysis or stability of the enzyme (Jung et al., 2016). Interestingly, Cys247 was found to be oxidized to cysteinyl sulfinic acid in the crystal structure, and this modification was proposed to be either catalytically relevant or to represent a non-productive damaged form of the protein (Pettinati et al., 2015). In 2016, Jung et al. showed that most cysteine residues in ETHE1 were endogenously polysulfidated by using a PEG-maleimide-based gel-shift assay and LC–MS/MS (Jung et al., 2016). They further demonstrated that the C247S mutant markedly reduced the polysulfidation levels, leading them to propose that polysulfidation of Cys247 and subsequent intramolecular sulfide transfer are important for regulating ETHE1 activity, although the mechanism has not yet been clarified in detail (Jung et al., 2016).

**FIGURE 12:**
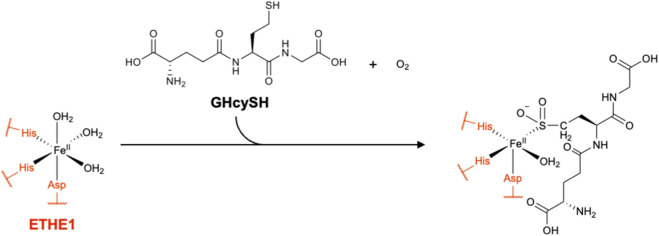
Inhibitor of ETHE1. Chemical structure of GHcySH and its inhibitory mechanism.

With respect to pharmacological tools, in 2018, Kabil et al. reported *γ*-glutamyl-homocysteinyl-glycine (GHcySH) as a mechanism-based ETHE1 inhibitor (Kabil et al., 2018). In this GSSH analog, the cysteinyl residue of GSH is replaced by homocysteine. ETHE1 recognizes GHcySH as an alternative substrate and oxidizes it to the corresponding sulfinic acid (GHcy-SO_2_H), which mimics the putative GSH perthiosulfinic acid (GS-SO_2_H) intermediate formed from GSSH (Figure 12). However, since GHcy-SO_2_H contains a C–S bond rather than an S–S bond, it cannot undergo the final hydrolytic step of the catalytic cycle and instead accumulates as a dead-end product, leading to time-dependent inactivation of the enzyme (Kabil et al., 2018). To date, GHcySH represents the best-characterized small-molecule probe for ETHE1. However, its use in cells or whole bodies has not yet been fully explored. Given that GHcySH is a close structural analogue of GSH, careful evaluation of potential off-target effects on other sulfur-metabolizing enzymes and GSH-processing enzymes, such as *γ*-glutamyl transpeptidase (Mitrić and Castellano, 2023), will be required.

### TST

TST, also known as rhodanese, is a mitochondrial matrix enzyme that participates in both cyanide detoxification and mitochondrial sulfide oxidation (Buonvino et al., 2022). Historically, TST was characterized in relation to the former process, in which it transfers a sulfur atom from thiosulfate to cyanide, producing thiocyanate and sulfite (Leininger and Westley, 1968; Way, 1984). Current understanding of this enzyme, at least under physiological conditions, is that it preferentially catalyzes sulfur transfer from GSSH to sulfite, producing thiosulfate and GSH in the mitochondrial sulfide oxidation pathway (Libiad et al., 2014; Buonvino et al., 2022; Libiad et al., 2015). In this context, TST acts downstream of SQOR and ETHE1, which generate GSSH and sulfite, respectively, and thereby contributes to mitochondrial H_2_S catabolism.

**FIGURE 13:**
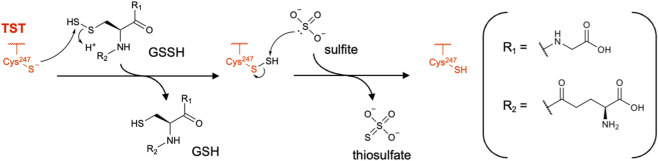
Proposed catalytic mechanism of TST.

The TST-catalyzed reaction follows a classical double-displacement (ping–pong) mechanism mediated by an active-site cysteine residue, similar to that of the 3MST-catalyzed reaction. In the first half-reaction, Cys248 attacks GSSH to form an enzyme-bound cysteine persulfide intermediate, releasing GSH. In the second half-reaction, a sulfite molecule attacks the terminal sulfur atom of this persulfide, yielding thiosulfate and regenerating free Cys248 at the active site (Figure 13) (Libiad et al., 2014; Buonvino et al., 2022; Libiad et al., 2015). In 2014, Libiad et al. reported a detailed kinetic analysis, showing that the *k*_cat_/*K*_m_ value for the TST-catalyzed reaction with GSSH as a sulfur donor and sulfite as a sulfur acceptor was nearly 1,000-fold higher than that for the reverse reaction with thiosulfate as a sulfur donor and GSH as a sulfur acceptor (Libiad et al., 2014). Structural characterization of bovine TST (rhodanese) has been extensively performed (Kruithof et al., 2020), and this bovine enzyme exhibits 90% amino acid sequence identity to human TST (Gliubich et al., 1998). In 2022, the first crystal structure of human TST was reported (Protein Data Bank (PDB) ID: 8AGF), and it aligns well with the bovine rhodanese structure, as expected from the high sequence similarity.

To date, no selective small-molecule inhibitor of TST suitable for mechanistic analysis in cells or tissues has been reported. Thiol-reactive reagents such as *N*-ethylmaleimide, iodoacetic/iodoacetamide derivatives and nitric oxide donors can inactivate TST by modifying the catalytic cysteine via *S*-alkylation or *S*-nitrosylation, but they are not specific to this enzyme (Buonvino et al., 2022; Wang and Volini, 1968; Horowitz and Criscimagna, 1982; Kwiec et al., 2003). Similarly, sodium 2-propenyl thiosulfate (2-PTS), a metabolite of allyl sulfur compounds, was shown to bind in the active site of *Azotobacter vinelandii* rhodanese (RhdA) and inhibit its activity (Sabelli et al., 2008). This compound acts as a thiosulfate analog and forms a 2-propenyl disulfide adduct on the active-site cysteine. Since RhdA shows similar kinetic behavior and high structural similarity to bovine rhodanese, 2-PTS is likely to inhibit the activity of human TST as well. The induction of apoptosis observed in cancer cells upon exposure to 2-PTS might be caused, at least in part, by inactivation of TST, though off-target effects cannot be excluded (Sabelli et al., 2008). 2-Methyl-1,4-naphthoquinone (menadione) is another compound reported to reduce the activity of TST in cells (Wróbel and Jurkowska, 2007). However, this compound reacts directly with GSH and H_2_S and likely causes the reactive oxygen species-mediated oxidation of cysteine residues in a non-specific manner (Mauzeroll et al., 2004; Croppi et al., 2020). Thus, no selective TST inhibitor for biological applications has yet been found.

## Summary and conclusion

Enzymatic generation and regulation of RSS are important for redox signaling, metabolic regulation and stress responses in mammalian systems. CBS, CSE, 3MST, CARS, SQOR, ETHE1 and TST work together as a network to maintain RSS homeostasis in cells (Figure 1). In this review, we have summarized structural and mechanistic studies to clarify how these enzymes work at the molecular level and have also described available inhibitors, focusing on their mechanisms and selectivity toward the target enzymes.

As discussed in the earlier sections of this review, the molecular mechanisms of the inhibitors have been proposed on the basis of the reaction kinetics and structural analyses. In particular, X-ray crystal structures of enzyme–inhibitor complexes provide direct evidence for elucidating the modes of inhibition. The enzyme–inhibitor complexes described in this review and deposited in the PDB are summarized in Table 1. Three of the five structures (CBS–AOAA, CSE–CPC and CSE–oxamic hydrazide) are oxime-, imine- or oxamic hydrazone-type complexes with PLP in the active site of CBS or CSE (Petrosino et al., 2022; Yadav et al., 2019; Echizen et al., 2023). One structure captures a covalent adduct at the active-site tyrosine residue (CSE–PAG) (Sun et al., 2009), and the remaining structure reveals a unique long-distance electrostatic interaction between the active-site cysteine persulfide anion and the positively charged carbonyl carbon of the inhibitor (3MST–I3MT-3) (Hanaoka et al., 2017). Given these structures and the stability of the oxime, *O*-alkyl and oxamic hydrazone complexes, AOAA, PAG and oxamic hydrazide are likely to inhibit their target enzymes in an irreversible manner. In contrast, CPC, which forms an imine (Schiff base) with PLP, has been reported as a reversible inhibitor of CSE (Yadav et al., 2019). I3MT-3, which binds to active-site residues in a non-covalent manner and shows a *K*_d_ value of 0.5 μM, is also considered a reversible inhibitor (Hanaoka et al., 2017).

**TABLE 1:** Enzyme–inhibitor complexes described in this review.

Enzyme	Inhibitor	PDB ID	Notes	References
CBS	AOAA	7QGT	PLP–oxime dead-end complex	Petrosino et al. (2022)
CSE	PAG	3COG	Covalent adduct on tyrosine (irreversible inhibitor)	Sun et al. (2009)
	CPC	6NBA	PLP-bound amino acrylate complex (reversible inhibitor)	Yadav et al. (2019)
	Oxamic hydrazide	8J6N	PLP–oxamic hydrazone	Echizen et al. (2023)
3MST	I3MT-3 (HMPSNE)	5WQK	Interacts with active-site cysteine persulfide	Hanaoka et al. (2017)

Extensive efforts have been undertaken to identify potent inhibitors of the enzymes involved in RSS biogenesis and regulation, using chemical synthesis and high-throughput screening (HTS) of large chemical libraries (Sasaki et al., 2023). As a result, numerous inhibitors targeting these enzymes have been reported. However, many of them are not highly selective, or their selectivity has not been sufficiently characterized, or they have not been applied to biological samples such as cells, tissues or animals. For example, even in the case of AOAA, historically the most commonly used CBS inhibitor, concerns have repeatedly been raised about its severe off-target effects on other PLP-dependent enzymes (Whiteman et al., 2011; Asimakopoulou et al., 2013; Zuhra et al., 2020). We summarize the current knowledge about key inhibitors of CBS, CSE, 3MST, CARS, SQOR, ETHE1 and TST discussed in this review in Table 2. Oxamic hydrazide, I3MT-3 and STI1 appear to be relatively reliable and selective inhibitors of CSE, 3MST and SQOR, respectively (Echizen et al., 2023; Hanaoka et al., 2017; Jackson et al., 2022). In contrast, no selective inhibitors are currently available for CBS or TST, and the selectivity of GHcySH for ETHE1 over other GSH-relating enzymes remains unknown. Moreover, despite the increasing number of studies demonstrating the biological importance of CARS (Nishimura et al., 2024; Zainol et al., 2023; Ogata et al., 2023), the molecular mechanism underlying its CPERS activity has not yet been elucidated, and no small-molecule inhibitor has been reported.

**TABLE 2:** Inhibitors of enzymes involved in RSS biogenesis and regulation.

Enzyme	Inhibitor	Type/mechanism	Selectivity/issues
CBS	AOAA	PLP-directed covalent inhibitor (oxime formation)	Broad-spectrum PLP inhibitor
	CH004	Competitive inhibitor	More selective than AOAA, but not CBS-specific
CSE	PAG	Mechanism-based irreversible inhibitor	Inhibits other PLP enzymes at higher doses
	CPC	Competitive inhibitor (cystathionine analog)	Limited off-target effects reported
	Oxamic hydrazide	PLP-dependent reversible inhibitor	Higher selectivity for CSE vs. other PLP enzymes
3MST	I3MT-3 (HMPSNE)	Persulfide-state–selective inhibitor	3MST-selective, known off-target inhibition to caspase-1
CARS	—	—	No CPERS-selective inhibitor reported
SQOR	STI1	CoQ-site competitive inhibitor	High selectivity among CoQ-dependent enzymes
ETHE1	GHcySH	Mechanism-based inhibitor (GSSH analog)	Potential off-target effects on GSH-utilizing enzymes
TST	—	—	No TST-selective inhibitor reported

Overall, the current series of inhibitors allow reasonably detailed studies of RSS-generating and regulating enzymes, but its scope remains insufficiently comprehensive. Selective CBS inhibitors with minimal off-target effects on other PLP-dependent enzymes, CPERS-specific CARS modulators, and highly selective ETHE1 and TST inhibitors have not yet been reported. Integrating chemical insights into the unique reactivity of persulfides and polysulfides with conventional HTS approaches should facilitate the development of more selective and efficient inhibitors and lead to a deeper understanding of RSS biology, as well as having the potential to provide novel therapeutic strategies for cancer, cardiovascular and metabolic diseases, and mitochondrial disorders in which RSS play a critical role.
